# Methyl Donor Supplementation Blocks the Adverse Effects of Maternal High Fat Diet on Offspring Physiology

**DOI:** 10.1371/journal.pone.0063549

**Published:** 2013-05-02

**Authors:** JesseLea Carlin, Robert George, Teresa M. Reyes

**Affiliations:** University of Pennsylvania, School of Medicine, Department of Pharmacology, Institute for Translational Medicine and Therapeutics, Philadelphia, Pennsylvania, United States of America; University of Otago, New Zealand

## Abstract

Maternal consumption of a high fat diet during pregnancy increases the offspring risk for obesity. Using a mouse model, we have previously shown that maternal consumption of a high fat (60%) diet leads to global and gene specific decreases in DNA methylation in the brain of the offspring. The present experiments were designed to attempt to reverse this DNA hypomethylation through supplementation of the maternal diet with methyl donors, and to determine whether methyl donor supplementation could block or attenuate phenotypes associated with maternal consumption of a HF diet. Metabolic and behavioral (fat preference) outcomes were assessed in male and female adult offspring. Expression of the mu-opioid receptor and dopamine transporter mRNA, as well as global DNA methylation were measured in the brain. Supplementation of the maternal diet with methyl donors attenuated the development of some of the adverse effects seen in offspring from dams fed a high fat diet; including weight gain, increased fat preference (males), changes in CNS gene expression and global hypomethylation in the prefrontal cortex. Notable sex differences were observed. These findings identify the importance of balanced methylation status during pregnancy, particularly in the context of a maternal high fat diet, for optimal offspring outcome.

## Introduction

Maternal nutrition during pregnancy represents a modifiable variable that has the potential to broadly affect fetal development. Excess energy intake, including increased fat intake, can lead to excessive maternal weight gain. A majority of women exceed the Institute of Medicine's (IOM) recommended guidelines for weight gain during pregnancy, with nearly 50% of women with a normal prepregnancy body mass index (BMI) and close to 75% of women with a high BMI (26–29 kg/m^2^) gaining more weight than recommended [1], [2]. Excessive gestational weight gain (GWG) increases adverse health outcomes for not only the mother, but for the child as well. Importantly, excessive GWG increases the risk for obesity in the offspring [3]–[7], a finding replicated in rodent offspring born to dams fed a high fat diet during pregnancy and/or lactation [8], [9].

Maternal consumption of high fat or high sucrose diets during pregnancy and/or lactation can affect brain development in the offspring. Neural circuitry that controls food intake is vulnerable to dysregulation, including neuropeptide systems within the hypothalamus that regulate homeostatic food intake [10], as well as neurotransmitters expressed within the mesocorticolimbic reward circuitry [11], which play a critical role in hedonically-driven feeding. Changes in reward-related neural circuitry influence behaviors, such as preference for sucrose, fat or palatable foods [11]–[14], which directly increase the risk for obesity, as these palatable foods are also calorie dense. The µ-opioid receptor (MOR) is expressed broadly throughout the central nervous system, and is a central player in coding the rewarding properties of natural stimuli such as palatable foods, as well as opiate drugs (e.g., morphine and heroin [15]) and nicotine [16] (also, see excellent, extensive recent review [17]). Activation of the MOR in the ventral striatum or cortex drives animals to selectively seek out fat-containing [18] or high carbohydrate foods [19], respectively, while a MOR inverse agonist attenuates the rewarding properties of sucrose [20]. Therefore, increased signaling through MOR may promote obesity by enhancing consumption of highly palatable (high fat, high carbohydrate), energy dense foods. Because of this, MOR is a target for a potential therapeutic directed towards obesity and disordered eating [21]. In addition to opioids, dopamine plays an important role in mediating reward-related behaviors, and there is a complex interplay between dopamine and MOR expression and function, with bi-directional regulation between the two systems [22]–[24].

Previously we identified increased expression of MOR and DAT mRNA, as well as both gene specific and global decreases in DNA methylation within the brains of male offspring born to HF fed dams [11]. The present experiments were designed to attempt to reverse this DNA hypomethylation through supplementation of the maternal diet with methyl donors, and to determine whether methyl donor supplementation could block or attenuate phenotypes associated with maternal consumption of a HF diet. Importantly, the present studies also extend our observations to include female offspring.

## Materials and Methods

### Animals and experimental model

All procedures were approved by the Institutional Animal Care and Use Committee (IACUC) of the University of Pennsylvania (protocol #801501). C57BL/6J virgin females were bred with DBA/2J males (F1 hybrid animals are used in all studies to allow for allele tracking done in related experiments, not reported here). All animals (males and females) were maintained on the control diet prior to breeding. Once bred, pregnant dams (n = 6/group) were fed one of four diets; (1) control diet, (2) high fat (HF) diet, (3) control +methyl donor supplementation (Control+Met) and (4) high fat +methyl donor supplementation (HF+Met). Formulated diets (Test Diet, Richmond, IN) were as follows; control diet (#5755) 18.5% protein, 12% fat and 69.5% carbohydrate and high fat diet (#58G9) 18.5% protein, 60% fat and 20.5% carbohydrate. The supplementation protocol involved the addition of the following to each kg of diet: 15 g Choline Chloride, 15 g Betaine (anhydrous), 15 mg Folic acid, 1.5 mg Vitamin B12, 7.5 g L-methionine, and 150 mg Zinc from ZnSO4·7H2O [25], which has previously been shown to alter DNA methylation patterns during development [26]. Dams were fed the diet from the onset of breeding, through pregnancy and lactation, and were maintained on their respective diets through the end of lactation in an effort to more fully cover the period of offspring brain development in a manner consistent with a full term human pregnancy [27]. Litters size did not differ across the groups, and at weaning, all offspring were maintained on the control diet. One animal per litter was used in individual experiments, to control for any litter effect. Male and female offspring were followed longitudinally and tested at the following time points (1) 12 and 20 weeks of age-metabolic assessments, (2) 40 weeks of age- fat and sucrose preference test, and (3) 50 weeks of age-brain collection for gene expression and methylation assays.

### Fat preference test

Fat preference: Mice were presented with both control diet and HF diet (60%) for 24 hrs on 3 separate test days. Intake of control diet, high fat diet and animal weight were measured. Intake was normalized to body weight and data from the final two days was averaged and analyzed (day one was excluded to allow animals to adapt to the novel food). Fat preference was calculated as a percentage of high fat diet consumed in relation to total food intake.

### Sucrose preference

For the duration of the test, offspring were caged individually and fed *ad libitum*. Mice were given simultaneous access to two bottles, one with water and one with 4% sucrose solution for 48 hours. Bottle order was random and was switched after 24 hours. Intake of consumed water, sucrose solution and animal weight were measured every 24 hours. Water, sucrose and the total fluid intake was normalized to body weight. Sucrose preference was calculated as a percentage of sucrose solution consumed in relation to total fluid intake.

### Metabolic measurements

Animals were tested in the Comprehensive Lab Animal Monitoring System (CLAMS, Columbus Instruments, Columbus, OH), which monitors food and water intake, indirect calorimetry, and x-axis activity. Animals had unrestricted access to powdered chow through a feeder located in the middle of the cage floor, which was directly connected to a precision scale. At least one week prior to testing, animals were housed in the CLAMS overnight to acclimate to powdered food and a novel cage. While in the CLAMS cages, animals had *ad libitum* access to powdered diet and water. Food intake (kcal) was normalized to body weight. For the 12 week assessment, animals were placed in the cages one hour prior to lights out and were removed 20 hrs later. Data from the 12 hr dark period were analyzed and reported. For the 20 week assessment, animals were housed in the metabolic cages for 44 hrs. Data from the two dark periods were averaged and reported.

### Genomic DNA and Total RNA isolation

Animals were euthanized with an overdose of carbon dioxide, followed by cervical dislocation; a method recommended by the Panel on Euthanasia of the American Veterinary Medical Association. Brains were then rapidly removed and placed in RNAlater (Ambion, Austin, TX) for 24 hours before dissection. Brain dissections to isolate the prefrontal cortex, the nucleus accumbens and the ventral tegmental area were preformed as previously described (Vucetic et al. 2010, Reyes et al. 2003, Cleck et al. 2008). Genomic DNA and total RNA were isolated simultaneously using AllPrep DNA/RNA Mini Kit (Qiagen).

### Gene expression analysis by quantitative Real-Time PCR

For each individual sample, 500 ng of total RNA was used in reverse transcription using High Capacity Reverse Transcription Kit (ABI, Foster City, CA). Expression of target genes was determined by quantitative RT-PCR using gene specific Taqman Probes with Taqman gene expression Master Mix (ABI) on the ABI7900HT Real-Time PCR Cycler. Gene probes are listed in supplemental material. Relative amount of each transcript was determined using delta CT values as previously described in (Pfaffl 2001). Changes in gene expression were calculated against the geometric mean of GAPDH and beta actin. Fold change values were calculated against the control+no supplement group. Primers used: Glyceraldehyde 3-phosphate dehydrogenase (GAPDH) Mm99999915_g1, Actin, beta (ACTB) Mm00607939_s1, preproenkephalin (PENK) Mm01212875_m1, opioid receptor, mu 1 (MOR) Mm01188089_m1, solute carrier family 6 (neurotransmitter transporter, dopamine) member 3 (DAT) Mm00438388_m1.

### Methylated DNA Immunoprecipitation (MeDIP) Assay

MeDIP assay was preformed using MagMeDIP kit (Diagenode, Denville, NJ). Methylated DNA was immunoprecipitated using 0.15 ul of magnetic beads coated with anti-5methylcytidine antibody (Diagenode) or mouse pre-immune serum. Enrichment in MeDIP fraction was determined by quantitative RT-PCR using ChIP-qPCR Assay Master Mix (SuperArray) on the ABI7900HT Real-Time Cycler. For all genes examined, primers were obtained from SuperArray (ChIP-qPCR Assays (−01) kb tile, SuperArray) for the amplification of genomic regions spanning the CpG sites located approximately 300–500 bp upstream of the transcription start sites. MeDIP results were expressed as fold enrichment of immunoprecipitated DNA for each site. To calculate differential occupancy fold change (% enrichment), the MeDIP DNA fraction CT values were normalized to Input DNA fraction CT values.

Primers used: OPRM1 Mouse Oprm1, NM_001039652.1 (-)01Kb: GPM1042701(-)01A, ACTIN Mouse Actb, NM_007393.2 (-)01Kb: GPM1051747(-)01A, SLC6A3 Mouse Slc6a3, NM_010020.3 (-)01Kb: GPM1031009(-)01A.

### Global Genomic DNA Methylation

Changes in genomic DNA methylation were analyzed using LUminometric Methylation Assay (LUMA). Methylation sensitive and insensitive restriction enzymes (HpaII and MspI, respectively) each digested 500 ng DNA with EcorI as an internal control (all enzymes from New England Biolabs, Beverly, MA) in Tango Buffer (33 mm Tris-acetate, pH 7.9, 10 mM Mg-acetate, 66 mM K-acetate, 0.1 mg/ml BSA) purchased from Fermentas (Fermendtas Scandanavia, Stolkholm) for 4 hours at 37 degrees Celcius. 20 ul of each digestion was mixed with 20 ul “Annealing Buffer” and transferred to Pyrosequencing plates. DNA quantification was performed using a polymerase extension assay by the Pyrosequencing tm platform. Pyromark Gold q96 Reagents and Pyromark q96 HS Plate was purchased from Qiagen. Peak height results were obtained to calculate methylation percentage as follows: [1-((HpaII/EcoRI)/(MspI/EcoRI))] ×100.

### Statistics

**Figure 1:**
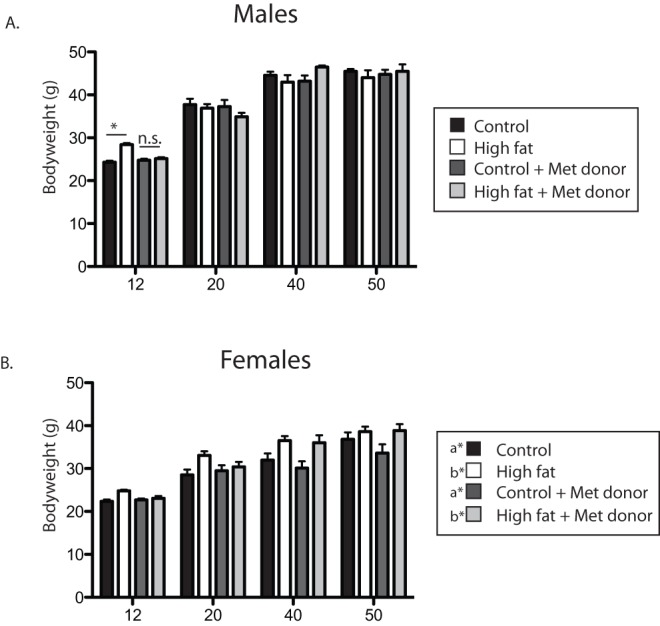
Body weight change over time. Body weights of male (A) and female (B) offspring were measured from 12–50 weeks of age. Male offspring from HF-fed dams (white bars) weighed significantly more than offspring from control fed dams (black bars) at 12 weeks of age, and this difference was normalized in offspring from methyl donor supplemented dams. Female offspring (B) from HF-fed dams (white bars) weighed significantly more than offspring from control fed dams (black bars) throughout the experiment. *p<0.05 (Bonferroni posthoc), *a and *b designate a main effect for maternal diet.

Two-way ANOVA (diet × supplementation) was used to evaluate results. Repeated measures by time were conducted for maternal weight gain during pregnancy (Table 1) and offspring weight gain over time (Fig. 1). Bonferroni posthoc comparisons were conducted when appropriate. Because, the primary goal was to determine whether methyl donor supplementation would block or reverse phenotypic changes driven by maternal consumption of the HF diet, and because methyl donor supplementation could have effects independent of the fat content of the maternal diet, inclusion of the control+Met group was a necessary control. Therefore, the comparisons of interest were; (1) control versus high fat offspring (to determine the effect of maternal HF diet), (2) control + methyl donor versus high fat + methyl donor offspring (to determine if methyl supplementation changed the effect of HF diet) and (3) control vs control + methyl donor (to determine if the methyl donor supplementation had an effect in control fed dams). Interactions and main effects are described in the results and significant posthoc comparisons are indicated in the figures. P≤0.05 was considered significant.

**Table 1:** Maternal weight during pregnancy (grams, mean (s.e.)) on days 4–17 post conception (n = 6/group). Average litter weight (g) and litter size (# pups).

Dam weight:	Control	High Fat	Control + methyl donor	HF + methyl donor
**Day 7**	19.04 (.33)	21.08 (.64)	19.66 (.34)	21.72 (.31)
**Day 14**	22.00 (1.28)	26.96 (.61)*	24.10 (.72)	25.96 (1.47)
**Day 17**	27.38 (2.37)	33.60 (.81)*	29.98 (1.09)	31.22 (2.97)
**Pup weight at birth** (range)	1.38 (.02) (1.14–1.57)	1.34 (.01) (1.17–1.51)	1.26 (.02)*^$^ (0.98–1.45)	1.35 (.02) (0.90–1.69)
**Average litter size**	6.75 (.48)	8.2 (.37)	7.2 (.66)	8.3 (1.03)

(*p<0.05, significantly different from control group, $p<0.05, significantly different from HF + methyl donor).

## Results

Analysis of maternal weight across pregnancy revealed a three way interaction (diet × supplement × time: F(2,14) = 4.63, p<0.03, Table 1). All dams gained weight during the pregnancy, and, posthoc analyses show that HF-fed dams weigh significantly more at d14 and d17 than control fed dams, whereas in the methyl donor supplemented groups there was no difference in maternal body weights between control and HF fed dams. A significant interaction was evident in the individual pup weights at birth (F(1,133) = 11.10, p = 0.001), as well as a main effect for supplementation (F(1,133) = 7.37, p = 0.008). Pups from the methyl donor supplemented dams weighed less than non supplemented pups, with the strongest effect in the control+methyl donor supplemented group. Litter size did not differ between the groups (Table 1). Pups remained with the dams and on their respective diets until weaning at 21 days of age, at which point all offspring were maintained on the control diet.

Body weight of the offspring was measured at 12, 20, 40 and 50 weeks of age, corresponding with the timepoints of the metabolic assessments (12 and 20 weeks), preference tests (40 weeks), and sacrifice (50 weeks). A two-way repeated measures ANOVA for the males revealed a diet x supplement x time interaction (F(3,18) = 4.84, p = 0.01). Posthoc analyses revealed that the only significant body weight difference was between control and HF-fed offspring at 12 weeks of age (a difference that was absent in the supplemented groups, Figure 1). In females, there was a main effect for maternal diet (F(1,19) = 23.65, p<0.0001), such that offspring from dams fed the HF diet were heavier, regardless of methyl donor supplementation status.

**Figure 2:**
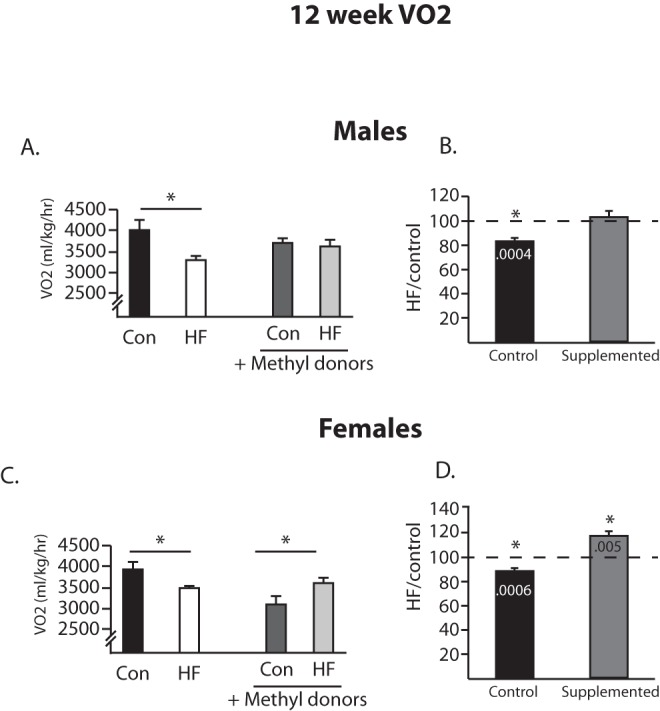
Metabolic rate at 12 weeks of age. Metabolic rate (oxygen consumption) at 12 weeks was measured in males (A, B) and females (C, D). Male offspring from HF-fed dams (white bars) have a significantly reduced metabolic rate (A) as compared to controls (black bars), and there is no difference in metabolic rate in the methyl donor supplemented groups (gray bars). Percent change analysis showed that in the unsupplemented groups, maternal HF diet leads to a significant decrease in VO2, while in the supplemented groups, these values do not differ (B). In females, offspring from HF-fed dams (white bars) have a significantly reduced metabolic rate (C) as compared to controls (black bars), and in the methyl donor supplemented groups, the offspring from HF-fed dams have a significantly increased metabolic rate (gray bars). This pattern is evident in the percent change analysis (D). *p<0.05 (Bonferroni posthoc), (actual p-values displayed in the bars in 1B, 1D). (n = 6/group).

Metabolic assessments (food intake, locomotor activity, and metabolic rate) were conducted at 12 and 20 weeks of age in male and female offspring. There were no differences observed in food intake. At 12 weeks of age, a 2×2 ANOVA revealed a significant interaction in the VO2 of male offspring (F(1,20) = 4.62, p = 0.04, Fig. 2A), such that maternal HF diet decreased VO2, and this difference was normalized in the methyl donor supplemented groups. To better understand how the methyl donor supplementation altered the response to the HF diet, percent change scores were calculated by dividing the VO2 of animals from HF fed dams by the average of the control offspring (for both unsupplemented and supplemented groups) and then testing whether that value was significantly different from 100% (e.g., values that are significantly different from 100%, either below or above, indicate an effect of the HF diet). In the unsupplemented groups, HF diet led to a significant decrease in VO2 in the male offspring (t_5_ = 8.53, Fig. 2B), and this effect was absent in the supplemented groups. In females, a significant interaction was again detected (F(1,20) = 14.66, p = 0.001, Fig. 2C), such that maternal HF diet decreased VO2, but the opposite pattern was observed in the supplemented groups. This is clear in the percent change analysis, where HF diet led to a significant decrease in VO2 in the unsupplemented groups (t_5_ = 7.64), however, in the supplemented groups, HF diet led to a significant increase in VO2 (t_5_ = 4.77, Fig. 2D). When assessed again at 20 weeks of age, there were no longer any significant differences observed for VO2 in either male or female offspring.

**Figure 3:**
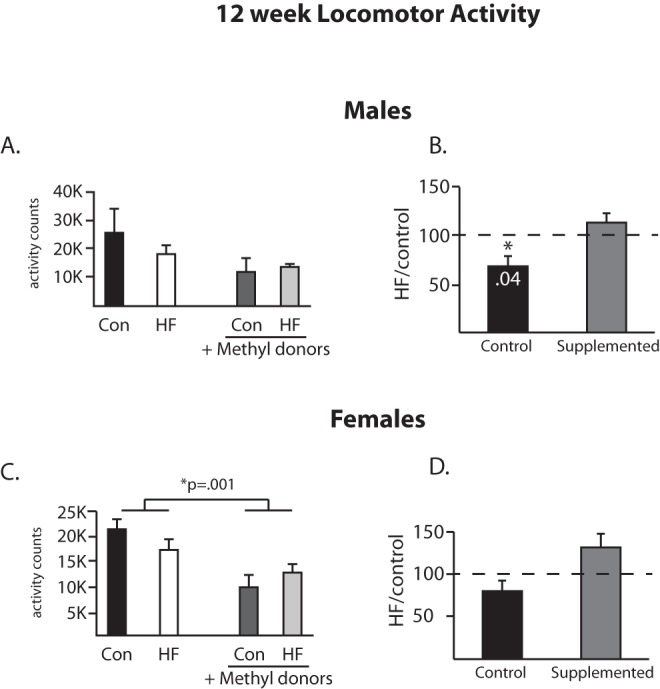
Locomotor activity at 12 weeks of age. Locomotor activity was measured at 12 weeks of age in males (A, B) and females (C, D). At 12 weeks of age, the percent change in locomotor activity as a result of maternal HF diet consumption is significantly decreased in the unsupplemented groups, and not different in the methyl donor supplemented groups (1B). In female offspring, the methyl donor supplementation led to a significant decrease in locomotor activity in females, regardless of the fat content of the maternal diet (1C). (n = 6/group).

**Figure 4:**
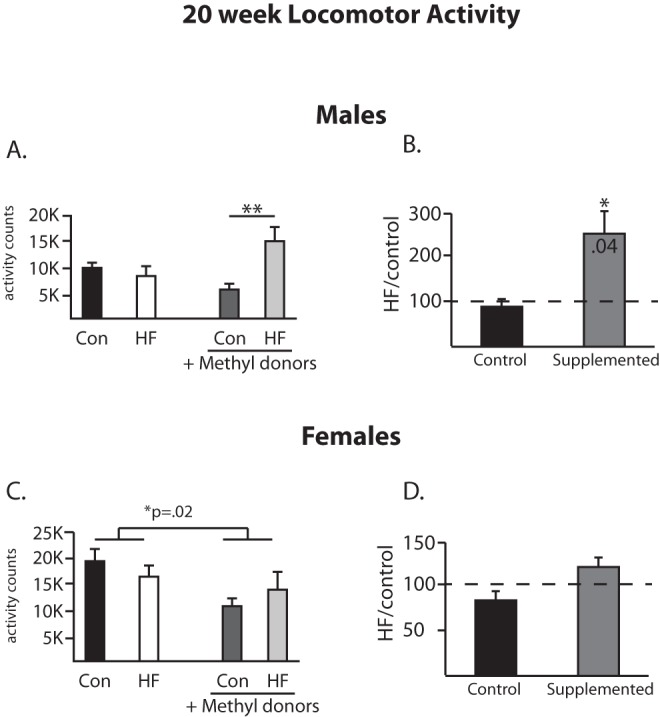
Locomotor activity at 20 weeks of age. Locomotor activity was measured at 20 weeks of age in males (A, B) and females (C, D). At 20 weeks of age, the percent change in locomotor activity as a result of maternal HF diet consumption is not changed in the unsupplemented groups, and in the methyl donor supplemented groups the animals from dams fed a high fat diet have a significant increase in locomotor activity (1B). In female offspring, the methyl donor supplementation led to a significant decrease in locomotor activity in females, regardless of the fat content of the maternal diet (1C). **p<0.01 (Bonferroni posthoc) (n = 6/group).

Locomotor activity was also evaluated. At 12 weeks of age, the 2×2 ANOVA revealed no significant differences between the groups in male offspring (Fig. 3A). The percent change analysis showed that the effect of the HF diet in the non-supplemented groups was to decrease activity (t_5_ = 2.6, Fig. 3B), with no difference observed in the supplemented animals. In females, the 2×2 ANOVA revealed a main effect for the methyl donor supplementation (F(1,19) = 14.2, p = 0.0013, Fig. 3C) such that the methyl donor supplementation of the dam decreased the overall level of activity in female offspring, regardless of the fat content of the maternal diet. At 20 weeks of age, a significant interaction was detected in the male offspring locomotor data (F(1,20) = 8.86, p = 0.0074, Fig. 4A). Analysis of the percent change values revealed that unsupplemented animals did not differ from each other regardless of the maternal diet, while in the context of the methyl donor supplementation, a maternal HF diet led to a significant increase in locomotor activity (t_5_ = 2.77, Fig. 4B) in male offspring. In females, a pattern identical to that seen at 12 weeks of age was observed, as the 2×2 ANOVA revealed a main effect for the methyl donor supplementation (F(1,20) = 6.3, p = 0.02, Fig. 4C) such that the methyl donor supplementation of the dam decreased the overall level of activity regardless of the fat content of the maternal diet.

**Figure 5:**
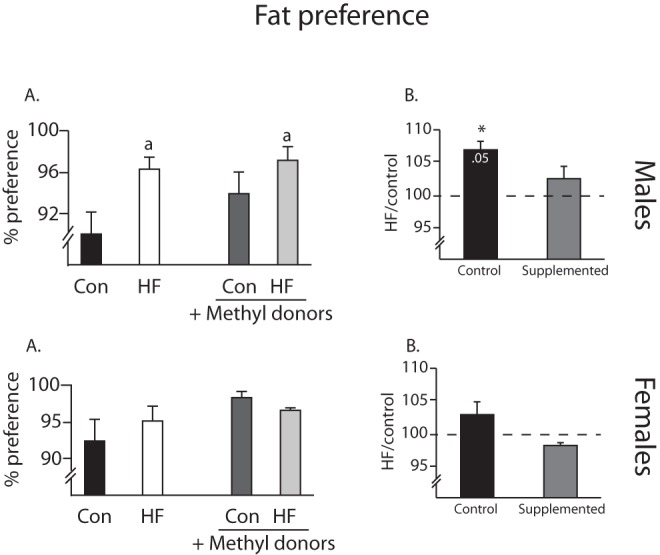
Fat preference. Males (top) from HF-fed dams (white bar) have a significantly increased preference for fat, as compared to control offspring (black bars), when tested at 40 weeks of age (main effect for maternal diet indicated by “a”). This difference in normalized in offspring from the methyl donor supplemented groups (gray bars). *p<0.05 (n = 6/group). There is no difference in fat preference in the females (bottom).

At 40 weeks of age, offspring were given a fat preference test, in which they were allowed to choose freely between control diet and HF diet. In male offspring, there was a main effect for diet, such that animals from dams fed a HF diet showed a significant increase in fat preference (F(1,20) = 7.31, p = 0.014, Fig. 5A). The percent change analysis showed that the effect of the HF diet in the non-supplemented groups was to increase fat preference significantly (t_5_ = 5.32, Fig. 5B), while in the supplemented group the increase was not significantly different from 100%. Contrary to the males, there were no differences in fat preference between the groups in female offspring. Sucrose preference tests were also performed and there were no differences in sucrose preference or water intake between the groups in either sex (data not shown).

**Figure 6:**
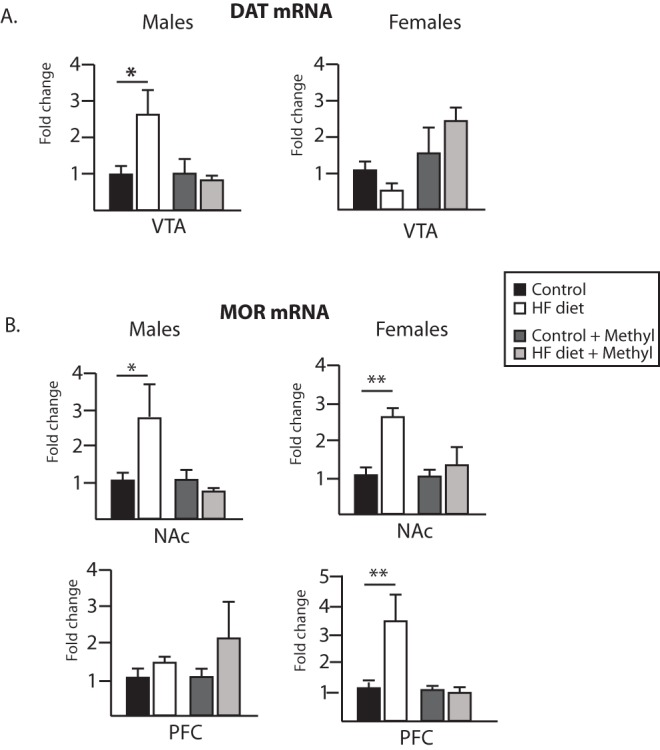
DAT and MOR mRNA expression is altered in the central reward circuitry in male and female offspring at 50 weeks of age. DAT mRNA (A) in the VTA is significantly increased in males from HF-fed dams. Maternal HF diet consumption leads to increased MOR mRNA (B) in the NAc (males and females) and PFC (females only). These mRNA changes are normalized in the methyl donor supplemented groups. *p<0.05, **p<0.01 (n = 5/group).

At 50 weeks of age, animals were sacrificed and gene expression was evaluated in the brain. DAT mRNA levels were examined in VTA in both male and female offspring (Fig. 6A). In males, ANOVA revealed a significant interaction (F(1,14) = 5.94, p = 0.03), such that male offspring from HF-fed dams showed a significant increase in DAT mRNA as compared to control offspring (p<0.05), a difference which was normalized in the methyl supplemented groups. In females, there were no significant differences in the expression of DAT mRNA.

MOR mRNA expression in the brains of the offspring was also determined. ANOVA analyses revealed a significant difference in MOR mRNA expression in both male and female offspring in the NAc (Fig. 6B). In males, a significant interaction was apparent (F(1,14) = 5.83, p = 0.03), with high fat diet increasing MOR expression only in the unsupplemented dams (p<0.05). Females showed the identical pattern, with a significant interaction (F(1,14) = 4.46, p = 0.05), such that maternal high fat diet increased MOR expression only in the unsupplemented dams (p<0.01). In females, but not males, this pattern was also observed in PFC (interaction; F(1,16) = 6.52, p = 0.02), with posthoc analyses supporting a significant increase only in offspring from maternal HF/no supplementation dams (p<0.01).

**Figure 7:**
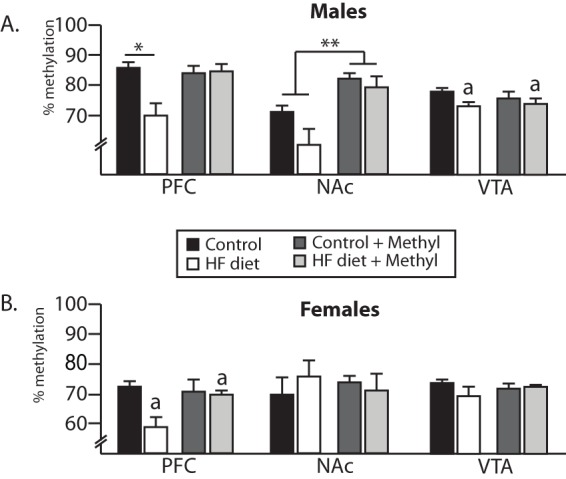
Changes in global DNA methylation in the PFC, NAc and VTA. In the male PFC, offspring from dams fed a HF diet showed global hypomethylation that was corrected in the supplemented groups. In the male VTA and female PFC, there was a significant main effect for maternal diet (regardless of supplementation status, designated with the letter “a”, p<0.05) and in the male NAc, there was a main effect for supplementation. *p<0.01, **p<0.001. (n = 5/group).

Global DNA methylation was evaluated in males and females, and there were notable differences across the brain regions. In both males and females, in the PFC, a similar pattern was observed, with high fat diet decreasing global methylation in the unsupplemented groups, an effect that was reversed with methyl donor supplementation (males, significant interaction; F(1,14) = 7.86, p = 0.01, female, main effect diet (F(1,15) = 5.3, p = 0.04, Figure 7). In the male NAc, there was a significant effect for supplementation (F(1,15) = 17.5, p = 0.0008), with supplementation increasing global methylation levels. In the VTA, a main effect for diet was observed (F(1,15) = 6.04, p = 0.026), with high fat diet decreasing global methylation in a similar manner regardless of supplementation status. In females, there were no differences across the groups in either NAc or VTA. We also investigated DNA methylation within the promoter region of the MOR in male and female offspring using a MeDIP assay. Results were variable, and there were no reliable differences in MOR promoter methylation in any brain region (data not shown).

## Discussion

The rationale for this study was our previous finding that maternal HF diet during pregnancy and lactation resulted in decreased global DNA methylation within the CNS [11]. This led to the hypothesis that supplementation of the maternal diet with methyl donors may reverse this global hypomethylation, and subsequently correct the adverse phenotypes associated with maternal high fat diet consumption during pregnancy. Maternal HF diet consumption during pregnancy and lactation led to phenotypes that contribute to an increase risk for offspring obesity, namely decreased metabolic rate (both sexes), increased fat preference (males) and gene expression changes within the CNS reward circuitry. Supplementation of the maternal diet with methyl donors attenuated or reversed the development of these adverse outcomes.

Maternal consumption of a high fat diet increased offspring body weight, and supplementation of the maternal diet with methyl donors blocked this response in males (12 weeks) and appeared to attenuate the early weight gain in females (12 and 20 weeks). Decreased metabolic rate and decreased locomotor activity contribute to positive energy balance and weight gain, and in these animals, decreased metabolic rate in particular appears to be affected by maternal high fat diet. Importantly, this is reversed by methyl donor supplementation to the pregnant dams. Additionally, the supplementation increased locomotor activity in males, which would also serve to limit weight gain, but not females (an important exception which will be discussed below). In a recent study, methyl donor supplementation (identical to the protocol used in the present studies) prevented the transgenerational amplification of obesity in obese A^vy^ mouse dams [28]. In that study, the underlying physiological mechanisms were not explored, and the present data would suggest that normalization of metabolic rate and/or effects on locomotor activity may in part, explain the prevention of obesity programming.

In addition to decreased metabolic rate, overconsumption of highly palatable, energy dense foods also contributes to an increased risk for weight gain/obesity (e.g., fat and sweet preference are positively correlated with overweight and obesity in adolescents [29]). Numerous reports in the literature have shown that offspring exposed to a high fat diet *in utero* or in early life have altered preference for fatty foods [12], [14], [30]. Additionally, male offspring from rat dams fed a high fat diet increased their operant responding for high fat pellets, in essence working harder than control animals for the fat pellet [13], with no difference in response rates for sucrose. Our previous study demonstrated an increase in fat preference and changes in gene expression in males at 20 weeks of age [11], and here these effects are shown to persist to 40–50 weeks of age. The addition of methyl donors to the maternal diet reduced the magnitude of the increase in fat preference in male offspring from HF fed dams. This is in line with similar findings in which folate supplementation was able to reverse an increased preference for high-fat food typically seen in the offspring born to a malnourished dams (low protein diet during pregnancy) [31]. For male offspring, maternal consumption of a HF diet increased MOR mRNA in the NAc, but not the PFC. Given that activation of the MOR in the ventral striatum drives animals to selectively seek out fat-containing foods [18], while activation of MOR in the cortex drives the selective consumption of high carbohydrate foods [19], this may partly explain why differences in fat preference were observed in the absence of an effect on sucrose preference. Circuitry changes within the hypothalamus, which are linked to the homeostatic control of food intake, are known to be affected by maternal obesity [32], [33], and our data extend these observations to highlight disruption of gene expression within the reward-related neural circuitry, important for hedonically-driven feeding (feeding beyond metabolic need).

Female offspring demonstrated robust changes in MOR mRNA expression, with increased expression in both NAc and PFC. There are a number of potential mechanisms whereby increased expression of MOR (and subsequent increased signaling through the receptor) will increase obesity risk, including increased preference for palatable foods (examined here), but also through increased motivation to work for palatable foods [20], [34]. It remains to be determined why females from HF-fed dams did not demonstrate an increased preference for fat, particularly given the increased expression of MOR in the NAc, and it will be interesting to examine their motivation to work for palatable food in future experiments. One explanation for the null finding may be increased variability in the females. Estrus cycle status was not controlled in these studies, and female hormones are known to affect fat intake [35]. Further, males have been shown to have a significantly stronger preference for fat than females [36], and it may have been necessary to examine a different percentage of fat to observe an effect in females. Consistent with known sex differences in DAT expression [37], DAT was unchanged in the females and this may partly explain the observed differences in fat preference. DAT in the VTA is expressed in neurons that project to both NAc and PFC. DAT clears dopamine from the synapse, so increased DAT in males would suggest less DA in the PFC and/or NAc. Dopamine is known to regulate the expression and function of the MOR, both *in vitro* [24] and *in vivo* [22], so differential levels of dopamine in males and females would affect MOR function and could contribute to the differential behavioral responses observed between the sexes. Importantly, in all instances in which mRNA levels were increased as a result of maternal HF diet, the methyl donor supplementation reversed this effect. As noted, promoter methylation of MOR and DAT did not differ between the groups. Supplementation with methyl donors will affect not only DNA methylation, but will alter histone methylation as well [38], therefore, methyl donor supplementation can affect gene expression without directly affecting DNA methylation within the proximal promoter region.

These data replicate our previous finding of global DNA hypomethylation within the reward circuitry of offspring born to dams fed a HF diet during pregnancy and lactation, and identify important sex (females are less affected) and regional (the PFC appears particularly vulnerable) differences in the CNS response to maternal high fat diet and methyl donor supplementation. This global DNA hypomethylation is also seen in response to other adverse early life conditions, such as environmental toxin exposure [39], nutrient restriction due to uterine artery ligation [40] or prenatal cocaine exposure [41]. Importantly, as predicted, this hypomethylation, particularly within the PFC, was reversed in both males and females when the maternal diet was supplemented with methyl donors. In the male NAc, supplementation led to increased methylation in both groups, while in the male VTA, the supplementation was ineffective in reversing the effect of maternal HF diet. The amount of global DNA methylation represents methylation status of repetitive DNA elements, transposons and intergenic sequences, which are typically heavily methylated. While loss of genomic DNA methylation contributes to locus- and gene-specific changes in DNA methylation, it also leads to broader effects such as chromosomal instability, aberrant activation of endogenous retroviral elements, and loss of imprinting [42] Little is known about the effects of global DNA hypomethylation in the CNS, however, forebrain-specific DNA methyltransferase 1 (*DNMT1*) knockout animals (which demonstrate global hypomethylation) provide some information. These animals have progressive neurodegeneration, altered neuronal gene expression, and severe degeneration of cortical neurons [43], [44]. Additionally, global DNA hypomethylation has been associated with neural tube defect affected pregnancies [45]. Research into the mechanisms and functional implications of differential global DNA methylation in the CNS is in its infancy and the present data provide important information regarding changes in global methylation as a result of maternal diet across both sexes as well as documenting the ability of methyl donor supplementation to prevent these changes.

While the primary endpoint of interest was to determine whether methyl donor supplementation could mitigate the effects of the HF diet, an important secondary analysis was to evaluate whether the methyl donor supplementation, independent of the high fat diet, had any effect on offspring physiology. Overall, the methyl donor supplementation affected primarily the *response* to a high fat diet. A notable exception, however, was the decrease in locomotor activity in female offspring born to methyl donor supplemented dams (regardless of the fat content of the diet), which could increase the risk for obesity development by promoting weight gain. Defining the underlying mechanism of this response will require future studies. This is particularly important, as pregnant women are counseled to maintain adequate folic acid intake to reduce neural tube defects and folic acid is a methyl donor. It is clear that pregnant women occupy the full range of the scale when it comes to folic acid status. Folic acid intake varies widely (with levels affected by maternal ethnicity, education, and socioeconomic status), and it remains the case that most nonpregnant women of childbearing age in the United States consume less than the recommended amount of folic acid [46]. However, there are also women taking in excessive amounts, possibly inadvertently. Folic acid is acquired through foods, either naturally occurring or in fortified foods. In one study, 80 to 95% of nonpregnant women greatly exceeded adequate levels of serum folate through dietary folate alone [47]. And similarly, 26% of women report folic acid intake in doses exceeding the recommended 400 µg/d, with ∼1% of women exposed to doses exceeding 1,000 µg/d (considered the tolerable upper limit (TUL) for folic acid) from fortified food alone [46]. In addition to dietary folate, approximately 2/3 of pregnant women take folic acid containing supplements [48]. In fact, 10% of pregnant women exceed the IOM recommended TUL only through supplementation [48]. Therefore, these multiple folate sources can combine to lead to an excess of methyl donors. The effects of supraphysiological doses of this methyl donor on the developing fetus and fetal brain specifically are unclear, but the present results would suggest that caution is warranted.

We identified two physiological responses that contribute to obesity risk in the offspring of dams fed a HF diet; (1) reduced metabolic rate and (2) increased fat preference coupled with increased expression of MOR within the reward circuitry of the brain. These changes represent a risk profile, and if placed into an environment in which highly palatable food was freely available, the prediction would be that these animals would gain even more weight than controls. Importantly, the methyl donor supplementation protocol used in the current study reversed changes in global DNA methylation in the offspring brain and reversed these phenotypes observed in response to maternal consumption of a HF diet during pregnancy and lactation. Further, the methyl donor supplementation alone had effects, specifically decreasing locomotor activity in female offspring. These data strongly support the importance of adequate and appropriate methyl donors in the perinatal diet, particularly in the context of a high fat diet. As prepregnancy BMI increases, the percentage of women not getting enough folate, which is a methyl donor, increases significantly [49]. Similarly, women with higher prepregnancy BMI have poorer diet, in part defined by the consumption of more fat and less folate [50]. In order to minimize the adverse outcomes of children born to mothers with excessive gestational weight gain and/or consumption of a high fat diet during pregnancy, establishment of optimal methyl status for pregnant women across the range of BMIs should be established.
